# Estimation of the effective reproduction number of influenza based on weekly reports in Miyazaki Prefecture

**DOI:** 10.1038/s41598-019-39057-w

**Published:** 2019-02-22

**Authors:** Takenori Yamauchi, Shouhei Takeuchi, Yuko Yamano, Yoshiki Kuroda, Toshio Nakadate

**Affiliations:** 1 Department of Hygiene, Public Health and Preventive Medicine Showa University, School of Medicine, Tokyo, Japan; 2 Department of Nutrition Science, Faculty of Nursing and Nutrition, University of Nagasaki, Nagasaki, Japan; 3 Department of Public Health, Faculty of Medicine, University of Miyazaki, Miyazaki, Japan

## Abstract

In Japan, as part of surveillance for seasonal influenza, the number of patients per influenza sentinel site is counted on a weekly basis. Currently, reference values are set for the weekly reported number of influenza cases per sentinel, and pre-epidemic and epidemic warnings are issued based on these values. In this study, we examined the association between these reference values and the effective reproduction number (*R*_t_) using surveillance data for Miyazaki Prefecture collected from 2010 to 2011. There are nine public health centre jurisdictions in this prefecture, and *R*_t_ exceeded 1.0 at the time when pre-epidemic warnings were issued in almost all the jurisdictions. Thus, it was indicated that the validity of the reference value was also high for influenza transmission. However, our results indicated the presence of secondary epidemic caused by infections originating both from other jurisdictions and inner jurisdictions, and it is occasionally not possible to evaluate the end of an epidemic in a jurisdiction using only the reference value of termination. It is necessary to establish new methods after considering the situation in the surrounding jurisdictions for more detailed epidemic predictions.

## Introduction

Seasonal influenza is a respiratory infectious disease caused by the influenza virus, and it occurs annually during winter months. In Japan, influenza is designated as a Category V infectious disease by the Act on the Prevention of Infectious Diseases and Medical Care for Patients with Infectious Diseases (the Infectious Diseases Control Law) executed in 1999. Based on this law, seasonal influenza is listed as a target illness of the National Epidemiological Surveillance of Infectious Diseases (NESID)^1^, and specific medical facilities such as influenza sentinel sites are expected to report the number of cases to public health centre (PHCs) weekly. Data tallied up in increments of PHC. The number of patients per influenza sentinel site under the jurisdiction of each PHC is publicly available on Public Health Institute websites. Data on the reporting criteria of influenza are also provided. The administrators of influenza sentinel sites are expected to notify cases every Monday if a physician has examined a patient or deceased individual with influenza-specific clinical signs and symptoms, if a patient is suspected to have influenza or if a patient exhibits all symptoms (i.e. sudden onset, high fever, upper respiratory tract inflammation, and general malaise or other systemic symptoms). Notification is also necessary if all four symptoms are not observed, but the presence of a viral antigen is detected by a rapid diagnostic kit using nasal cavity aspirate, nasal cavity swab, and throat swab. Reporting criteria for sentinel hospitals are separately stipulated^2^.

Seasonal influenza is a threat to public health, and issuing of pre-epidemic and epidemic warnings can prevent potential epidemics. A pre-epidemic warning signifies that a massive epidemic can occur within the next four weeks, while an epidemic warning is issued when there are suspicions that a massive epidemic is ongoing or has been persisting. Pre-epidemic and epidemic warnings are basically issued in PHC units, contributing to the early grasp of infection spread and provision of a heads-up to PHC jurisdictions.

Pre-epidemic warnings are issued when the weekly reported number of influenza cases per sentinel (Weekly CPS) in a jurisdiction of PHC exceeds 10.0. The reference value of 10.0 was determined based on some probabilities, such as sensitivity (i.e. the probability that a pre-epidemic warning is issued within four weeks before the epidemic warning), specificity (i.e. the probability that no pre-epidemic warning is issued based on a false suspicion of an epidemic occurrence in the future) and positive predictive value (i.e. the probability that an epidemic warning is issued within four weeks after the pre-epidemic warning), which were 0.60–0.70, 0.95–0.98 and 0.20–0.30, respectively, over the last five years^3^. Epidemic warnings are issued when Weekly CPS in a PHC jurisdiction exceeds 30.0. If an epidemic warning has been issued in the past week, it will not be cancelled until the value is lower than 10.0. The reference value for epidemic warnings was also set according to a 1% probability for the occurrence of a series of epidemic warnings in the past five years^3^.

Meanwhile, the effective reproduction number (*R*_t_) is defined as the average number of secondary cases produced by a typical case in that particular population^4^. When *R*_t_ is higher than 1.0, the number of cases is expected to increase. However, it is expected to decrease when *R*_t_ is lower than 1.0. Therefore, *R*_t_ is considered useful in the evaluation of the transmissibility of infectious diseases^5^. However, few studies have reported on the association between *R*_t_ and pre-epidemic or epidemic warnings. At present, the reference value of the pre-epidemic and epidemic warning are set based on Weekly CPS, without considering the characteristic of the infectious disease. One of our aims was to confirm the consistency between the two evaluations on the epidemic (i.e. the *R*_t_ based evaluation considering factors associated with the influenza epidemic such as human movement, and the evaluation based on current reference value). We also aimed to clarify why secondary epidemics occur by estimating *R*_t_. In this study, a secondary epidemic is defined as a series of infected events, in which Weekly CPS exceeds 10.0 again after it had decreased to below 10.0. This is because the pre-epidemic warning is issued when Weekly CPS is more than 10.0, and the epidemic warning ends when it becomes less than 10.0.

## Results

### Descriptive epidemiological data of each PHC jurisdiction

**Figure 1:**
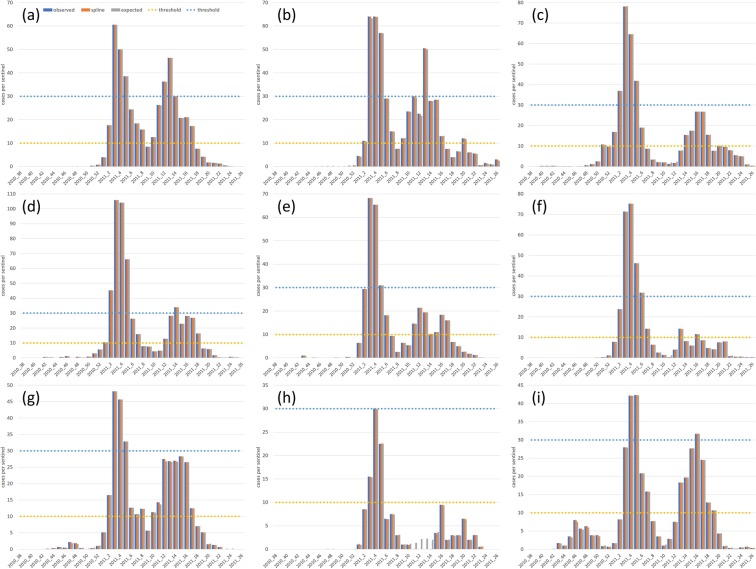
Observed and estimated epidemic cases by a spline and mathematical model. The weekly number of reported cases per sentinel (blue bar) as well as cases per sentinel estimated using a spline model (orange bar) and cases per sentinel estimated according to a mathematical model assuming the validity of the spline model (grey bar) are shown. The thresholds both for pre-epidemic (10.0) and epidemic warning (30.0) are also shown in yellow dotted line and blue dotted line, respectively. Pre-epidemic and epidemic warnings are issued when Weekly CPS exceeds 10.0 and 30.0, respectively. Epidemic warnings are cancelled when Weekly CPS is lower than 10.0. Cases in Miyazaki city, Chuo, Miyakonojo, Nobeoka, Nichinan, Kobayashi, Takanabe, Takachiho, and Hyuga are shown in (**a**–**i**), respectively.

During the 2010–2011 influenza season in Miyazaki Prefecture, the first case was reported in the Miyakonojo PHC; Weekly CPS in Miyakonojo was 0.3 at the 40^th^ week in 2010 (Fig. 1). In all the PHC jurisdictions, one or more phases in which Weekly CPS shifted from a decrease to an increase were observed, and a secondary epidemic defined in the introduction section was confirmed without Takachiho. According to a national population census conducted in 2010, Table 1 shows the population of each jurisdiction and the estimated number of people who moved between jurisdictions. The outflow population was large in the order of Takanabe, Miyakonojo, and Miyazaki city, and inflow population was in the order of Miyazaki city, Miyakonojo, and Takanabe. The ratio of population influx and efflux to the night-time population was large in the order of Takanabe, Chuo, and Hyuga with respect to influx and Chuo, Takanabe, and Kobayashi with respect to efflux. The epidemic ended in the 40^th^ week of 2010, in which Weekly CPS was higher than 0 at least in one jurisdiction. Similarly, the epidemic started in the 27^th^ week of 2011, and Weekly CPS became 0 in all the jurisdictions. Before the start of the epidemic, Weekly CPS continued to be 0 for two consecutive weeks from the 39^th^ week in 2010, and also continued to be 0 for two consecutive weeks till the 26^th^ week in 2011 after the end of the epidemic. Therefore, we evaluated the predictability of the spline model according to the Akaike’s information criterion (AIC), which had the highest applicability when the beginning and end of the epidemic were set at the 38^th^ week of 2010 and the 26^th^ week of 2011, respectively.

**Table 1:** The inflow and outflow population of each public health centre jurisdiction. The inflow and outflow populations from other jurisdictions are indicated in columns and rows, respectively. They were estimated by a national population census conducted in 2010.

	Miyazaki city	Chuo	Miyakonojo	Nobeoka	Nichinan	Kobayashi	Takanabe	Takachiho	Hyuga	From other prefecture	Total influx	population
Miyazaki city		2,415	3,484	1,350	1,191	2,873	4,026	928	3,099	1,404	20,770	401,138
Chuo	1,234		1,183	5	0	5	1,827	7	92	20	4,373	26,951
Miyakonojo	3,143	869		1,386	732	1,369	3,477	34	2,784	5,032	18,826	190,433
Nobeoka	939	97	907		8	24	2,718	4	1,089	402	6,188	125,159
Nichinan	27	14	624	14		3	2,034	4	39	354	3,113	72,869
Kobayashi	1,780	251	1,217	19	9		2,813	15	764	731	7,599	75,059
Takanabe	3,004	1,855	3,537	1,259	888	2,867		409	3,168	144	17,131	101,901
Takachiho	399	0	151	7	1	3	733		21	125	1,440	20,588
Hyuga	2,292	547	2,636	8	1	635	3,123	2		187	9,431	89,971
To other Prefectures	1,549	66	3,723	566	1,413	665	322	404	326		9,034	3,985,185
Total efflux	14,367	6,114	17,462	4,614	4,243	8,444	21,073	1,807	11,382	8,399		
population	401,138	26,951	190,433	125,159	72,869	75,059	101,901	20,588	89,971	3,985,185		

### Weekly *R*_t_ and its 95% credible interval

**Figure 2:**
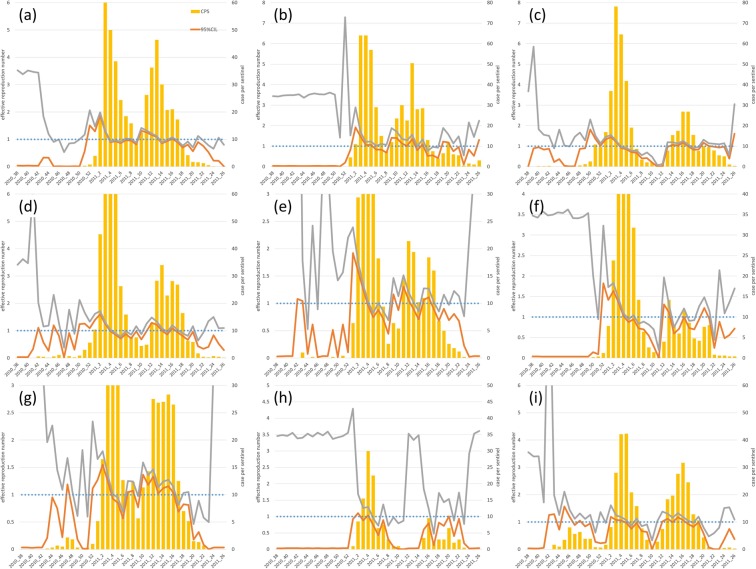
Observed epidemic cases and effective reproduction number. The weekly number of reported cases per sentinel (bar plot), the lower 95% credible interval limit of the effective reproduction number (*R*_t_) (95% CIL) (orange line), the upper 95% credible interval limit of *R*_t_ (95% CIU) (grey line) and the threshold both for *R*_t_ (1.0) and pre-epidemic warning (10.0) are shown in blue dotted line. The situations in Miyazaki city, Chuo, Miyakonojo, Nobeoka, Nichinan, Kobayashi, Takanabe, Takachiho, and Hyuga are shown in (**a**–**i**), respectively.

The *R*_t_ of each of the nine jurisdictions was estimated and plotted with Weekly CPS (Fig. 2). The 95% credible intervals of *R*_t_ were: Miyazaki city (1.9–2.0), Chuo (1.9–2.9), Miyakonojo (1.4–1.5), Nobeoka (1.4–1.6), Nichinan (1.6–1.8), Kobayashi (1.7–1.8), Takanabe (1.5–1.8), Takachiho (0.9–1.3), and Hyuga (1.1–1.2) when the pre-epidemic warning at each jurisdiction was issued, and Weekly CPS was higher than 10.0. Therefore, all the lower 95% credible interval limits were higher than 1.0, except that for Takachiho at the time of issual of the pre-epidemic warning. All the *R*_t_ values were lower than 1.0 when Weekly CPS value was lower than the reference value for the end of the epidemic warning (i.e. 10.0). Furthermore, once Weekly CPS was lower than 10.0, it exceeded 10.0 again in all the jurisdictions except Takachiho. Particularly, in Miyazaki city, Chuo, and Nobeoka, the value reached 30.0 again which is the reference value for epidemic warnings. In the case of a secondary epidemic, all the lower 95% credible interval limits were also higher than 1.0 except in Takachiho.

### Status of viral type detection

**Figure 3:**
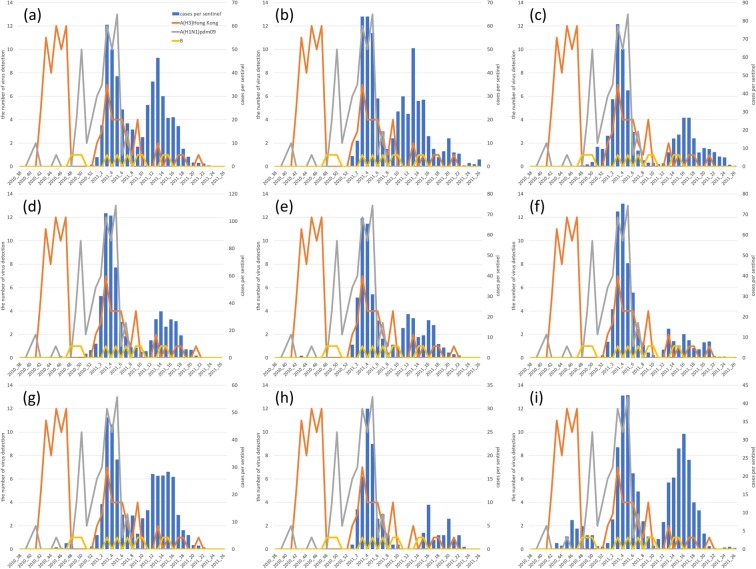
Observed epidemic cases and detected viral types. The weekly number of reported cases per sentinel is shown using bar plot. The number of A(H3)Hong Kong, A(H1N1)pdm09 and B cases are depicted using orange, grey, and yellow lines, respectively. The situations in Miyazaki city, Chuo, Miyakonojo, Nobeoka, Nichinan, Kobayashi, Takanabe, Takachiho, and Hyuga are shown in (**a**–**i**), respectively.

According to the surveillance conducted by the Public Health Institute (PHI) in the Miyazaki Prefecture from 38^th^ to 52^th^ (2010)^6^ week and from 1^st^ to 27^th^ week (2011)^7^, both type A and type B cases were prevalent during the influenza season from 2010 to 2011. The number of virus detections on the prefecture level and weekly reported cases per sentinel are shown in Fig. 3 since the virus types and the detected jurisdiction were noted until the 51^st^ week of 2010, and no jurisdiction has been noted after that. Although that surveillance did not provide a complete census, A(H3)Hong Kong were dominant until the 47^th^ week in 2010, and infections in Takanabe and Hyuga in 2010 were probably due to this virus type. A(H1N1)pdm09 was detected only in Miyakonojo until the 49^th^ week in 2010, and it was indicated that A(H1N1)pdm09 spread from Miyakonojo. Both A(H1N1)pdm09 and A(H3)Hong Kong were prevalent in the first epidemic, though A(H1N1)pdm09 was not detected after 8^th^ week in 2011. Therefore, it is reasonable that the secondary epidemics in all jurisdictions except Takachiho were mainly caused by A(H3)Hong Kong. Type B virus was sporadically detected throughout the influenza season from 2010 to 2011, but the association between the epidemic and type B virus was not clearly estimated.

### Ratio of infection from other jurisdictions to infection inside each jurisdiction

**Figure 4:**
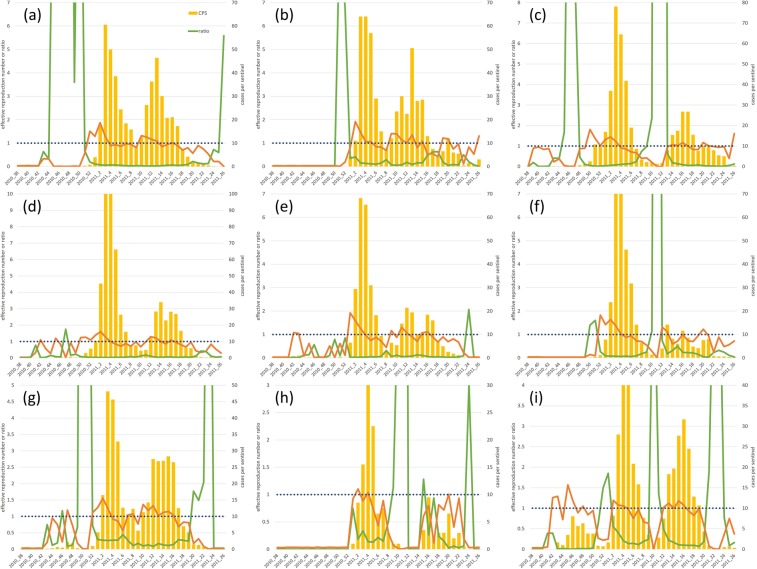
Observed epidemic cases and ratio of infections transmitted from other jurisdictions to infections within each jurisdiction. The weekly number of reported cases per sentinel (bar plot), the lower 95% credible interval limit of the effective reproduction number (*R*_t_) (95% CIL) (orange line) and the ratio of the expected value of daily cases of infection transmitted from other jurisdictions to that of infection within each jurisdiction (green line) are shown. The situations in Miyazaki city, Chuo, Miyakonojo, Nobeoka, Nichinan, Kobayashi, Takanabe, Takachiho, and Hyuga are shown in (**a**–**i**), respectively.

The lower limit of the 95% credible interval of *R*_t_ was higher than 1.0 at all jurisdictions without Takachiho in the week of secondary epidemics when Weekly CPS was initially over 10.0. The ratio of the expected value of daily cases of infections from other jurisdictions to that of infections within each jurisdiction was evaluated to estimate the contribution of internal and external infections (namely those prevalent within and outside a particular jurisdiction, respectively) (Fig. 4). Consequently, in Miyakonojo, Kobayashi, and Hyuga, the ratio was higher than 1.0 in the early stage of the secondary epidemic. In Miyakonojo, Weekly CPS was higher than 1.0 at the 14^th^ week in 2011, and the ratios at the 10^th^, 11^th^, and 12^th^ week were 2.4, 97.0, and 105.3, respectively. In Kobayashi, Weekly CPS was higher than 10.0 at the 13^th^ week in 2011, and the ratios at the 10^th^ and 11^th^ week were 1.1 and 56.2, respectively. In Hyuga, Weekly CPS was higher than 10.0 at the 13^th^ week in 2011, and the ratios at the 10^th^ and 11^th^ week were 7.7 and 1.4, respectively. The ratio was always lower than 1.0 from the first epidemic to the secondary epidemic in other jurisdictions except Takachiho, where no secondary epidemic was observed. Thus, it was indicated that there were two types of secondary epidemics. Infections from other jurisdictions contributed more strongly to secondary epidemics at Miyakonojo, Kobayashi and Hyuga.

### The goodness of fit to Weekly CPS

Weekly CPS, estimated value obtained by spline, and expected value obtained from the mathematical model were used to compare the weekly reported number of influenza cases per sentinel, as shown in Fig. 1. When a chi-square test of goodness of fit was conducted^8,9^, the deviation between Weekly CPS and estimated value was not significant (*P* < 0.05) at all jurisdictions. The estimated value and expected value were in good agreement in any jurisdiction.

## Discussion

In this study, the *R*_t_ was estimated using Weekly CPS of the influenza epidemic from 2010 to 2011 in Miyazaki Prefecture. Despite the deviation from their original definition, pre-epidemic warnings were issued when *R*_t_ exceeded 1.0, and the timing of pre-epidemic warning is probably reasonable even when the features associated with influenza epidemic such as human movement and serial interval were considered. It was also indicated that inter-jurisdictional infections triggered secondary epidemics in some jurisdictions.

In Takachiho, two specific features were observed. One was that the lower 95% credible interval limits of *R*_t_ was not higher than 1.0 when the pre-epidemic warning was issued. Weekly CPS in Takachiho was over 10.0 at the 3rd week in 2011, and it was under 10.0 at the 8th week in 2011. The maximum value of Weekly CPS was 30.0, and it was the least among the nine jurisdictions. Furthermore, the number of influenza-associated medical facilities in Takachiho was also the lowest among the nine jurisdictions, and the estimated number of influenza patients was also the lowest. At the 3rd week in 2011, the lower 95% credible interval limits were higher than 1.0 in all jurisdictions except Takachiho. Therefore, it was likely that infection from other jurisdictions could cause the infection in Takachiho, and *R*_t_ in Takachiho at the 3rd week in 2011 could have been underestimated. The other feature was that the secondary epidemic was not observed in Takachiho. One possible reason for this might be the lowest ratio of population influx and efflux to the night-time population. It was possible that it might have been difficult for the secondary epidemic to occur since there might have been be a lower risk of infection from other jurisdictions.

Both A(H1N1)pdm09 and A(H3)Hong Kong were prevalent in first epidemic, and only A(H3)Hong Kong was mainly prevalent in the secondary epidemic. However, the items to be notified at influenza sentinel sites are surveillance period, name of medical facility, sex, and age^10^. Because the serotype of the influenza virus is not included, it is not clarified in the weekly report. In Japan, type A cases often prevail prior to type B cases, but sometimes, they occur at almost the same time like in the case of the 2010–2011 epidemic. According to the Implementation manual for the National Epidemiological Surveillance of Infectious Diseases Program, sentinel sites for laboratory-based surveillance are to send at least one specimen per reporting interval. If information on serotype and strain is quantitatively clarified by appropriate sampling, it may be easier to more accurately predict transmission among jurisdictions by comparing the viruses prevailing in each jurisdiction. However, it is essential to carefully examine and evaluate the cost and benefits associated with the aggregation and identification of a viral serotype or strain.

Nishiura *et al*. investigated the association between reporting and serial intervals, and concluded that an ideal reporting interval is the mean serial interval^11^. Because the serial intervals of seasonal influenza, A(H1N1)pdm09 and type B range from 2 to 4 days^12–18^, *R*_t_ can be estimated with high accuracy if the reporting interval is set at approximately 3 days. Because weekly reports are aggregated in units of seven days, the average serial interval is greatly exceeded. Additionally, Nishiura *et al*. also proposed a correction method for estimating *R*_t_ in cases in which the reporting interval is not consistent with the serial interval. However, we focused not only on *R*_t_ estimation but also on the movement of populations and the consequent movement of infections between jurisdictions, and we used different models in this study. In the model adopted, data regarding the number of infected cases per day are necessary. Because the cumulative cases obtained by weekly reports during the epidemic roughly followed a cubic function, we estimated the number of infected cases per day by applying a cubic spline function as in a previous study^19^. Moreover, because there were jurisdictions in which Weekly CPS and estimated value by the spline function deviated from each other, it was impossible to deny the existence of a possible bias associated with the estimated value. However, the observed and the estimated values were well-fitted, and the use of the spline function in this study was not necessarily denied.

In Japan, the NESID has been conducted since 1999, and previous related studies have been reported. Murakami *et al*. analysed epidemic data from 1993 to 1997 in older regimes and suggested that the criteria for the start and end of epidemics in influenza-like disease was 30.0 and 10.0, respectively^20^. These criteria were re-evaluated in the year 2007 by Murakami *et al*., and they confirmed the presence of a few problems even if they were not modified^21^. However, in the influenza epidemic in Miyazaki Prefecture from 2010 to 2011, the epidemic occurred again even if Weekly CPS became lower than the reference value of 10.0 for the end of issuing the epidemic warning. In Miyazaki city, Chuo, Miyakonojo, Nobeoka, Nichinan, Kobayashi, Takanabe, and Hyuga, the numbers of weeks in which Weekly CPS was lower than 10.0 from the beginning of the first epidemic to the second pre-epidemic warning were 1, 1, 7, 4, 4, 5, 1, and 5 weeks, respectively. Our results indicated that there were two types of secondary epidemic defined in this study. One was caused by the infections from other jurisdictions, as was observed in Miyakonojo, Kobayashi, and Hyuga. In these cases, pre-epidemic warning effectively functioned because *R*_t_ was over 1.0 when Weekly CPS was over 10.0 again. However, to predict epidemics with high accuracy in a jurisdiction, the epidemic of neighbouring jurisdictions should be closely monitored. In other jurisdictions without Takachiho, secondary epidemics were mainly caused by the infections from the inner jurisdiction, and it was indicated that the first epidemic had not ended though Weekly CPS became lower than 10.0. There are several hypotheses to explain this. It is likely that the specific virus type was prevalent in the first epidemic, and infection with another virus type spread right after the first epidemic. In line with Murakami *et al*. who suggested the possibility that the local influenza epidemic would expand^22^, it is also possible that the distribution of influenza sentinel sites or patients did not have the representative necessary for predicting the epidemic, and Weekly CPS based prediction did not function well enough. In any case, it was suggested that the reference value based on Weekly CPS for the end of epidemic was not necessarily appropriate, and that there can be a limit to the prediction of the end of epidemic by CPS.

In 2004, Murakami *et al*. reviewed pre-epidemic warnings against influenza-like diseases. They concluded that the sensitivity, specificity, and positive predictive value were 90.4, 93.7, and 23.9%, respectively, and that the validity of a reference value of 10.0 for pre-epidemic warning was high^23^. Additionally, in this study, *R*_t_ exceeded 1.0 at the time of the issue of the pre-epidemic warning report, and we believe that new method of epidemic evaluation needs to be considered while setting the reference value for pre-epidemic warnings. Although the definition of the warning report is as described in the introduction, it was indicated that pre-epidemic warnings are useful as indices of the time to implement countermeasures against a future epidemic.

Our study has four major limitations partly at least due to data availability. First, the population influx and efflux among jurisdictions could have been over-estimated because the data were not summarized for a single PHC unit in the national population census though the number of people moving between cities is considered. Therefore, it is possible that the movement of populations from one jurisdiction to another could include the movement of populations from one city included in a jurisdiction to another city belonging to the same jurisdiction. However, in line with this, *R*_t_ in each jurisdiction may be under-estimated, and the conclusions of this study would not have been substantially altered. Second, analyses were conducted without identification of the viral serotype or strains because of the nature of NESID described above. The third limitation was the availability of public daily data. To our knowledge, there were no publicly available data on daily cases in each jurisdiction and on daily human movement. Thus, any difference between the movement or cases on weekdays and on weekends was not considered in this study. Consequently, there was no other way but to conduct estimations based on an approximated value. However, Weekly CPS could be assumed to have a representative as the data of the week because the pre-epidemic and the epidemic warning are originally to be issued based on Weekly CPS. One of our aims was to confirm the consistency between the two evaluations of the epidemic (i.e. *R*_t_ based evaluation and that based on Weekly CPS). Therefore, *R*_t_ was assumed to be the weekly altered index in this study. At the same time, if the movement on weekend greatly contributed to the epidemic, Weekly CPS and the weekly *R*_t_ might reflect the situation to some extent. To investigate the representativeness of weekly based evaluation, difference between weekly and daily based evaluation has to be compared in future studies. The last limitation is that no difference between clinics and hospitals was considered in this study. It is likely that more patients are examined in hospitals compared with clinics. However, according to the Implementation manual for the National Epidemiological Surveillance of Infectious Diseases Program, patient sentinel sites are supposed to be determined in order to be able to grasp the status of infectious diseases in the entire prefecture as much as possible, considering the distribution of population and medical institutions. Furthermore, the pre-epidemic and epidemic warnings were issued based on Weekly CPS, which is calculated by dividing the number of weekly reported cases by the number of influenza sentinel sites, with no coefficient reflecting the types of influenza sentinel sites. For our aim, although bias could not be ruled out, the impact of the bias would not be very large.

In conclusion, pre-epidemic warnings based on the current influenza surveillance system effectively functioned at least in the Miyazaki Prefecture. Considering the original definition of the pre-epidemic warning, the evaluation based on Weekly CPS was consistent with the evaluation based on *R*_t_, including features of influenza. The timing of pre-epidemic warning was probably appropriate. However, the reference value for the end of the epidemic remains to be improved mainly because it did not necessarily reflect the epidemic of inner jurisdiction, at least in Miazaki prefecture. It is essential to evaluate the epidemic situation considering all the available information on the infectious disease, such as viral serotype, viral strain, and the epidemic situation in the surrounding jurisdiction. It is also necessary to establish a methodology to judge the end of the epidemic in future studies.

## Methods

As influenza epidemic data, we used Weekly CPS at a PHC jurisdiction (*V*_*i*_(*w*)) from the 38^th^ week in 2010 to the 26^th^ week in 2011 in Miyazaki Prefecture^6,7^. Weekly CPS at one jurisdiction is calculated by dividing the number of weekly reported cases by the number of influenza sentinel sites. There are nine PHCs (i.e. Miyazaki city, Chuo, Miyakonojo, Nobeoka, Nichinan, Kobayashi, Takanabe, Takachiho, and Hyuga) in Miyazaki Prefecture. Here, *i* denoted nine jurisdictions and the imaginary “other prefecture”, which was defined as the prefecture with the average features of Kagoshima, Kumamoto, and Oita Prefecture (i.e. index *i* was varied from 1 to 10). Additionally, *w* was the index representing a week. The number of influenza-associated medical facilities [i.e. clinics and hospitals which included patients from the internal medicine or paediatrics departments as the examination subjects] (*h*_*i*_) in each PHC jurisdiction was obtained via data from the Kyushu Okinawa hospital, from 2001 to 2009. In this study, the number of clinics used within each jurisdiction was 232, 15, 95, 58, 42, 45, 61, 10 and 35. Then, the number of weekly influenza patients at each PHC jurisdiction was estimated by the following equation (1):

1 $${I}_{i}(w)={V}_{i}(w)\times {h}_{i},$$

On the other hand, the *V*_*i*_(*w*) outside Miyazaki Prefecture was defined as the average of Weekly CPS in Kagoshima, Kumamoto, and Oita prefectures as obtained by the NESID. We defined the *h*_*i*_ outside Miyazaki as the average number of medical facilities (i.e. all those clinics and hospitals which included patients from the internal medicine or paediatrics departments as the examination subjects) in each of the three prefectures as obtained by a medical facilities survey in 2010. The *I*_*i*_(*w*) outside Miyazaki Prefecture was estimated by equation (1). Finally, the number of daily influenza cases at each PHC jurisdiction ($\widehat{{I}_{i}}(t)$) was estimated as in the case of a previous study^19^ in which *t* was the index representing day. Briefly, a smoothing spline was fitted to the weekly cumulative curve of each jurisdiction. Then, the daily difference of the cumulative counts was taken in order to obtain the number of daily patients. In this process, if the estimated $\widehat{{I}_{i}}(t)$ was lower than 0, it was set at 0. It was also rounded to make it an integer. To obtain a coefficient of determination *R*^2^ at 0.995, the smoothing spline was used in this study. The spline model was evaluated by the AIC to obtain the most predictive model when the beginning and end of the epidemic varied from the 38^th^ week to the 44^th^ week in 2010 and from the 26^th^ week to the 28^th^ week in 2011.

The night-time population in a national population census in 2010 was defined as the population in each jurisdiction. Human movement between jurisdictions was also quantified by a national population census conducted in 2010. The census was summarized in a unit of city, town, or village in Miyazaki Prefecture, and each jurisdiction of PHC included more than one city, town, or village. Thus, municipal night-time, day-time, and inflow and outflow populations were tallied for each jurisdiction, and we estimated the number of people who moved from *j* to *i* (*c*_*ij*_), where *j* also denoted nine jurisdictions and the imaginary “other prefecture”. Data on efflux and influx of population between a jurisdiction in Miyazaki Prefecture and another prefecture (i.e. Kagoshima, Kumamoto, or Oita Prefecture) as well as vice versa were not fully represented in the national population census. Thus, the population of an imaginary “other prefecture” was defined as the average population of Kagoshima, Kumamoto, and Oita Prefecture.

Consequently, we modified the model reported previously^24^, and the expected value of daily cases (${\rm{E}}(\widehat{{I}_{i}}(t))$) was estimated by equations (2) and (3):

2 $${\hat{{\rm{\Lambda }}}}_{i}(t)={R}_{i}(t)\sum _{\tau =1}^{T}\,\omega (\tau ){\hat{I}}_{i}(t-\tau ),$$

3 $$\hat{E}(\widehat{{I}_{i}}({\rm{t}}))=f({c}_{ii})\hat{{{\rm{\Lambda }}}_{i}}(t)+\sum _{i\ne j}\,f({c}_{ij})\hat{{{\rm{\Lambda }}}_{j}}(t),$$

in which *f*(*c*_*ij*_), *ω*(*τ*), and *T* represent the rate of population who moved from *j* to *i* to the night-time population in *j*, the distribution of serial intervals *τ* days ago, and the cut-period for the serial interval distribution, respectively. We used shifted gamma distribution^25^ with a mean of 2.6 days and a standard deviation of 1.5 days^12,26^ as the serial interval distribution. Further, we assumed that the *R*_*i*_(*t*) value was the same during a given week. Each parameter was estimated by Markov chain Monte Carlo methods, assuming that *I*_*i*_(*t*) is Poisson-distributed with a mean of $\hat{E}({I}_{i}({\rm{t}}))$. The number of iterations, chains, and burn-in were 20,000, 8, and 5,000, respectively. In addition, the Rhat statistic of Gelman-rubin was determined to converge, due to the fact that it is less than 1.1. A sensitivity analysis of *R*_*i*_(*t*) was conducted by varying the mean and the standard deviation of serial interval distribution from 2.6 to 3.7 considering viral type A and B and from 1.5 to 2.0, respectively. In this study, the model uncertainty derived from serial interval was only considered, and that derived from interpolation of smoothing spline was not. The latter uncertainty is likely to be less influential on *R*_*i*_(*t*) since all coefficients of determination *R*^2^ for estimates by the smoothing spline were over 0.995 and the goodness of fit was not significant in any jurisdictions. The original model was used to analyze the spatial distribution of the Ebola epidemic. However, the only parameters that characterize the Ebola epidemic are serial interval and human movement parameters. In this study, we used parameters suitable for influenza, to investigate the spatial distribution of influenza infection among ten regions.

## Data Availability

A national census data was available from “https://www.e-stat.go.jp/stat-search/database?page=1&layout=datalist&toukei=00200521&tstat=000001080615&cycle=0&tclass1=000001101935&survey=%E5%9B%BD%E5%8B%A2%E8%AA%BF%E6%9F%BB&result_page=1&second=1&second2=1”. NESID data in Miyazaki Prefecture was available from “https://www.pref.miyazaki.lg.jp/contents/org/fukushi/eikanken/center/infectious/2010/index.html” and “https://www.pref.miyazaki.lg.jp/contents/org/fukushi/eikanken/center/infectious/2011/index.html”; those in other prefectures were available from https://idsc.niid.go.jp/idwr/CDROM/Kako/H22/SyuList.html” and “https://www.niid.go.jp/niid/ja/all-surveillance/2270-idwr/nenpou/3359-syulist2011.html”. Data regarding the number of medical institutions were available from “https://www.e-stat.go.jp/stat-search/files?page=1&layout=datalist&tstat=000001030908&cycle=7&tclass1=000001038857&tclass2=000001038860&second2=1”. However, the Kyushu Okinawa hospital data were not available free of cost and had to be purchased. Furthermore, the total number of clinics in each jurisdiction in the Miyazaki Prefecture has been shown in the Methods section.

## References

[1] Taniguchi K (2007). Overview of infectious disease surveillance system in Japan, 1999-2005. J. Epidemiol..

[2] Ministry of Health, Labour and Welfare. *About notification by medical doctor and veterinary doctor based on Act on the Prevention of Infectious Diseases and Medical Care for Patients with Infectious Diseases*, https://www.mhlw.go.jp/bunya/kenkou/kekkaku-kansenshou11/01-05-28.html (2018).

[3] National Institute Of Infectious Diseases. *About the system of issuing the pre-epicemic and epidemic warnings*, https://nesid4g.mhlw.go.jp/Hasseidoko/Levelmap/flu/guide.html (2007).

[4] De Serres, G., Gay, N. J. & Farrington, C. P. Epidemiology of transmissible diseases after elimination. *Am*. *J*. *Epidemiol*. **151**, 1039–1048, discussion 1049–1052 (2000). 10.1093/oxfordjournals.aje.a010145 10873127

[5] Wallinga J, Teunis P (2004). Different epidemic curves for severe acute respiratory syndrome reveal similar impacts of control measures. Am. J. Epidemiol..

[6] Miyazaki prefectural institute for public health and environment. *Miyazaki prefecture infectious disease weekly report back issue* (2010) https://www.pref.miyazaki.lg.jp/contents/org/fukushi/eikanken/center/infectious/2010/index.html (2010).

[7] Miyazaki prefectural institute for public health and environment. *Miyazaki prefecture infectious disease weekly report back issue* (2011) https://www.pref.miyazaki.lg.jp/contents/org/fukushi/eikanken/center/infectious/2011/index.html (2011).

[8] Favier C (2006). Early determination of the reproductive number for vector-borne diseases: the case of dengue in Brazil. Trop. Med. Int. Health.

[9] Chowell G, Nishiura H, Bettencourt LM (2007). Comparative estimation of the reproduction number for pandemic influenza from daily case notification data. J. R. Soc. Interface.

[10] Ministry of Health, Labour and Welfare. *National Epidemiological Surveillance of Infectious Diseases* (*Influenza sentinel sites*), https://www.mhlw.go.jp/bunya/kenkou/kekkaku-kansenshou11/pdf/01-07-02.pdf (2018).

[11] Nishiura H, Chowell G, Heesterbeek H, Wallinga J (2010). The ideal reporting interval for an epidemic to objectively interpret the epidemiological time course. J. R. Soc. Interface.

[12] Ferguson NM (2005). Strategies for containing an emerging influenza pandemic in Southeast Asia. Nature.

[13] Cowling BJ, Fang VJ, Riley S, Malik Peiris JS, Leung GM (2009). Estimation of the serial interval of influenza. Epidemiology.

[14] Levy JW (2013). The serial intervals of seasonal and pandemic influenza viruses in households in Bangkok, Thailand. Am. J. Epidemiol..

[15] White LF, Pagano M (2008). Transmissibility of the influenza virus in the 1918 pandemic. PLoS One.

[16] Cauchemez S (2009). Household transmission of 2009 pandemic influenza A (H1N1) virus in the United States. N. Engl. J. Med..

[17] Lessler J (2009). Outbreak of 2009 pandemic influenza A (H1N1) at a New York City school. N. Engl. J. Med..

[18] Yang Y (2009). The transmissibility and control of pandemic influenza A (H1N1) virus. Science.

[19] Nishiura, H. & Chowell, G. Early transmission dynamics of Ebola virus disease (EVD), West Africa, March to August 2014. *Euro Surveill*. **19** (2014). 10.2807/1560-7917.es2014.19.36.20894 25232919

[20] Murakami Y, Hashimoto S, Taniguchi K, Fuchigami H, Nagai M (2000). Temporal and geographical variation in epidemics determined from the results of an infectious disease surveillance system in Japan description of epidemic patterns by data-based criteria for epidemic periods. Nihon Koshu Eisei Zasshi.

[21] Murakami Y (2007). The impact of changing critical values of the early epidemic detection system for infectious disease surveillance in Japan. Nihon Koshu Eisei Zasshi.

[22] Murakami Y (2007). Wide-area epidemics of influenza and pediatric diseases from infectious disease surveillance in Japan, 1999-2005. J. Epidemiol..

[23] Murakami Y (2004). Evaluation of a method for issuing warnings pre-epidemics and epidemics in Japan by infectious diseases surveillance. J. Epidemiol..

[24] Backer JA, Wallinga J (2016). Spatiotemporal Analysis of the 2014 Ebola Epidemic in West Africa. PLoS Comput. Biol..

[25] Cori A, Ferguson NM, Fraser C, Cauchemez S (2013). A new framework and software to estimate time-varying reproduction numbers during epidemics. Am. J. Epidemiol..

[26] Boelle PY, Ansart S, Cori A, Valleron AJ (2011). Transmission parameters of the A/H1N1 (2009) influenza virus pandemic: a review. Influenza Other Respir Viruses.

